# Experimental Evaluation on the Heating Efficiency of Magnetoferritin Nanoparticles in an Alternating Magnetic Field

**DOI:** 10.3390/nano9101457

**Published:** 2019-10-14

**Authors:** Huangtao Xu, Yongxin Pan

**Affiliations:** 1 Biogeomagnetism Group, Paleomagnetism and Geochronology Laboratory, Key Laboratory of Earth and Planetary Physics, Institute of Geology and Geophysics, Chinese Academy of Sciences, Beijing 100029, China; xuhuangtao@mail.iggcas.ac.cn; 2 Institutions Earth Science, Chinese Academy of Sciences, Beijing 100029, China; 3 College of Earth and Planetary Sciences, University of Chinese Academy of Sciences, Beijing 100049, China

**Keywords:** magnetic nanoparticles, magnetic hyperthermia, magentoferritin, Néel relaxation, specific loss power, intrinsic loss power

## Abstract

The superparamagnetic substance magnetoferritin is a potential bio-nanomaterial for tumor magnetic hyperthermia because of its active tumor-targeting outer protein shell, uniform and tunable nanosized inner mineral core, monodispersity and good biocompatibility. Here, we evaluated the heating efficiency of magnetoferritin nanoparticles in an alternating magnetic field (AMF). The effects of core-size, Fe concentration, viscosity, and field frequency and amplitude were investigated. Under 805.5 kHz and 19.5 kA/m, temperature rise (ΔT) and specific loss power (SLP) measured on magnetoferritin nanoparticles with core size of 4.8 nm at 5 mg/mL were 14.2 °C (at 6 min) and 68.6 W/g, respectively. The SLP increased with core-size, Fe concentration, AMF frequency, and amplitude. Given that: (1) the SLP was insensitive to viscosity of glycerol-water solutions and (2) both the calculated effective relaxation time and the fitted relaxation time were closer to Néel relaxation time, we propose that the heating generation mechanism of magnetoferritin nanoparticles is dominated by the Néel relaxation. This work provides new insights into the heating efficiency of magnetoferritin and potential future applications for tumor magnetic hyperthermia treatment and heat-triggered drug release.

## 1. Introduction

Iron-oxide magnetic nanoparticles (MNPs) have been widely used in various biomedical applications, including tumor detection, imaging and therapy because of their excellent magnetic, optic and electric properties, biocompatibility and biodegradability [1,2,3]. In particular, MNPs show promising applications in magnetic hyperthermia therapy (MHT) for cancer treatment as an individual treatment or an adjuvant treatment combined with chemotherapeutic and/or radiotherapeutic agents [4,5]. It has been shown that MNPs can convert the magnetic energy into thermal energy through hysteresis loss or Néel/Brownian relaxation process in an alternating magnetic field [6,7,8]. For superparamagnetic iron oxide nanoparticles, the energy conversion is mostly based on relaxation process [9]. To distinguish the relaxation process between Néel relaxation and Brownian relaxation, a gel or glycerol system which has the different viscosity was usually used [10,11]. Alternatively, comparing the fitted effective relaxation time (obtained by SLP vs. frequency fitting) with Néel relaxation time and Brownian relaxation time (obtained by calculation) has been proposed [12].

In order to achieve a higher thermal energy intra tumor, MNPs in delivery systems for targeting deep or multiple tumor are requested [13]. To this end, linking ligands are applied, but it makes the synthesis process more tedious and ultimately results in lower productivity with higher cost. Over the past decade, a few cage-like proteins have been used for synthesis of nanosized magnetic minerals inside their cavity [14]. One of these MNPs is magnetoferritin, consisting of a recombinant ferritin shell and a magnetite inner core [15,16]. Outer protein shell ferritin composed of human heavy-chain ferritin has shown a great ability to actively targeting lots of tumors in vivo and in vitro through the transferrin receptor 1 which is over expressed on most tumor cell membrane [17,18,19]. The concept of using magnetoferritin for MHT was proposed by Babincová [20]. Recently, Fantechi and co-workers reported that a 6.8 nm sized and 5% Co-doped iron oxide core of magnetoferritin killed ~70% B16 melanoma cells in vitro by MHT [21]. Massner and co-workers have shown that magnetoferritin with 4 nm iron oxide core caused ~10% mortality of human embryonic kidney 293T cells in vitro [22]. Nevertheless, the heating efficiency and influencing factors of magnetoferritin in aqueous solution have not been studied in detail yet. In the present work, we carried out a systematic study to investigate the influences of core-size, Fe concentration, AMF amplitude and frequency on the specific loss power (SLP) and intrinsic loss power (ILP) of magnetoferritin in aqueous. We also measured the heating efficiency in media of different viscosity (glycerol-water mixture) to identify the heat generation mechanisms of magnetoferritin.

## 2. Materials and Methods

### 2.1. Preparation of Recombinant Human Ferritin (HFn)

The *Pichia pastoris* X-33 containing recombinant plasmid pPICZ A-HFn was constructed in the Beijing Biogeomagnetic Laboratory (Beijing, China). The expression of HFn was determined according to the EasySelect^TM^ Pichia Expression Kit user’s manual (Invitrogen, Carlsbad, CA, USA).

### 2.2. Synthesis of Magnetoferritin (MHFn)

The MHFn nanoparticles were synthesized according to our previous work [23]. Briefly, a deoxygenated solution of 0.1 M NaCl (40 mL) with HFn (0.5 mg/mL) was injected into a reaction vessel, kept the temperature at 65 °C and pH 8.5. Freshly prepared Fe (II) (25 mM (NH_4_)_2_Fe(SO_4_)_2_‧6H_2_O) and H_2_O_2_ (8.3 mM) were added to the vessel in a rate of 50 Fe/(protein‧min). To prepare MHFn nanoparticles with different mineral core sizes, different theoretical amounts of iron atoms per protein cage were added into the reaction vessel. The whole reactions were under an anaerobic atmosphere to the end. The synthesized MHFn nanoparticles were centrifuged to remove the aggregated nanoparticles. Finally, the MHFn nanoparticles were kept in an aqueous medium for further use.

### 2.3. Mineral Core Characterization

To characterize the mineral core’s size and crystallinity, the mineralized MHFn nanoparticles in aqueous medium were dried on an ultra-thin amorphous-carbon film and analyzed by transmission electron microscopy (TEM, JEOL JEM-2100, Tokyo, Japan) with an accelerating voltage of 200 kV. The inner core size of MHFn for each sample was averaged from about 1000 separated particles. Here, we measured the area of the single grain and then calculated the diameter with D = (4‧area/π)^1/2^. High-resolution TEM (HR-TEM) images and selected area electron diffraction (SAED) patterns were taken to analyze the crystallinity of mineral core of MHFn nanoparticles.

### 2.4. Magnetic Measurements of MHFn Nanoparticles

MHFn nanoparticles were freeze dried with an Alpha 1–2 LD plus freeze dryer (Martin Christ Gefriertrocknungsanlagen GmbH, Osterode am Harz, Germany). The dried samples were placed in a non-magnetic capsule with a copper spoon. The measurements were performed on a Quantum Design MPMS SQUID magnetometer (model XP-XL5, with magnetic moment sensitivity of 5.0 × 10^−10^ A‧m^2^, Quantum Design North America, San Diego, CA, America). Briefly, the hysteresis loops were measured in fields of ± 3T at 5 K and 300 K, respectively. Zero-filed cooled (ZFC) and filed-cooled (FC) curves were measured in a filed of 1.5 mT from 5 K to 200 K, the blocking temperatures (T_B_) was determined from the maximum of the ZFC curves. The acquisition of isothermal remanent magnetization (IRM) and dc demagnetization curves were measured in a fields of 0–1 T at 5 K.

### 2.5. Dynamic Light Scattering (DLS) Measurements and Thermogravimetric (TG) Analysis

The hydrodynamic diameter of the samples in aqueous medium were measured by dynamic light scattering (DynaPro NanoStar, Wyatt Technology Corporation, Goleta, CA, USA). Samples (10 μL) at 1.5 mg[Fe]/mL were added to the holder and tested three times. Thermogravimetric analysis (TG/DTA 6300, Seiko instruments Inc., Chiba, Japan) was used to obtain the proportion of the inner mineral core of the entire MHFn nanoparticles. Samples were heated from room temperature to 800 °C at 10 °C/min under an air flow at 1 L/min.

### 2.6. Heating Efficiency Analyses

The heating efficiency of MHFn samples have been measured with a commercial system DM100 series (nB nanoScale Biomagnetics, Zaragoza, Spain) at Shanghai Normal University (Shanghai, China). A MHFn aqueous sample (0.5 mL) in a 2 mL glass chromatography vial was set in the middle of the coil. The temperature of all samples during magnetic treatment was recorded by an optic fiber temperature probe with a response temperature of 0.1 °C. The initial temperature of each sample has been controlled and stabilized to room temperature (about 22–23 °C controlled by air condition).

Two different test methods were used to evaluate repeatability of samples under H = 19.5 kA/m and *f =* 808.5 kHz at 5 mg[Fe]/mL. One of them, named single test method, is one by one test after cooling to room temperature and measured four times, the other named continuous test method is to turn off the AMF when temperature rise to 33 °C and turn on the AMF when temperature is low to 28 °C for four cycles. The effects of size and concentration on SLPs and ILPs have been measured at H = 19.5 kA/m and *f =* 808.5 kHz with 1.5 and 5 mg[Fe]/mL MHFn samples. The dependences of AMF parameters on SLPs have also been investigated. Two core sizes of MHFn samples at 5 mg[Fe]/mL were tested at 805.5 kHz with different AMF amplitudes (11.9, 13.9, 15.9, 17.9, 19.5 kA/m) and at 19.5kA/m with different frequencies (274.5, 405.5, 466.0, 546.5, 598.0, 737.0, 805.5 kHz), respectively. 4.3 nm MHFn sample at 2.5 mg[Fe]/mL and 4.8 nm MHFn sample at 1.5 mg[Fe]/mL in different glycerol-water mixtures (0, 50% and 70% glycerol ratio) were also measured. In this work, we set the moment that field amplitude reached the setting value as t = 0, and set the temperature (about 22–23 °C) at t = 0 as ∆T = 0.

The method proposed by Kallumadil and co-workers was used to calculate the heating efficiency [24]: fitting the curves of field applied time vs. temperature with Box-Lucas equation [$T\left(t\right)=a\left(1-e^{-\mathrm{bt}}\right)$] to calculate the ∆T/∆t at t = 0; the heating efficiency SLP was calculated with $\;\mathrm{SLP}=C_{water}/c_{Fe}\times \frac{\Delta T}{\Delta t}$, where *C_water_* is the heat capacity of water (4.185 J‧g^−1^‧K^−1^) and *c_Fe_* is the Fe concentration in aqueous solution; the intrinsic loss power (ILP) was calculated by ILP = SLP/(*f*‧H^2^). The Néel-Brownian relaxation time and the effective relaxation time were calculated with formula given by Rosensweig [6].

## 3. Results and Discussion

### 3.1. Core Size and Crystallinity

**Figure 1:**
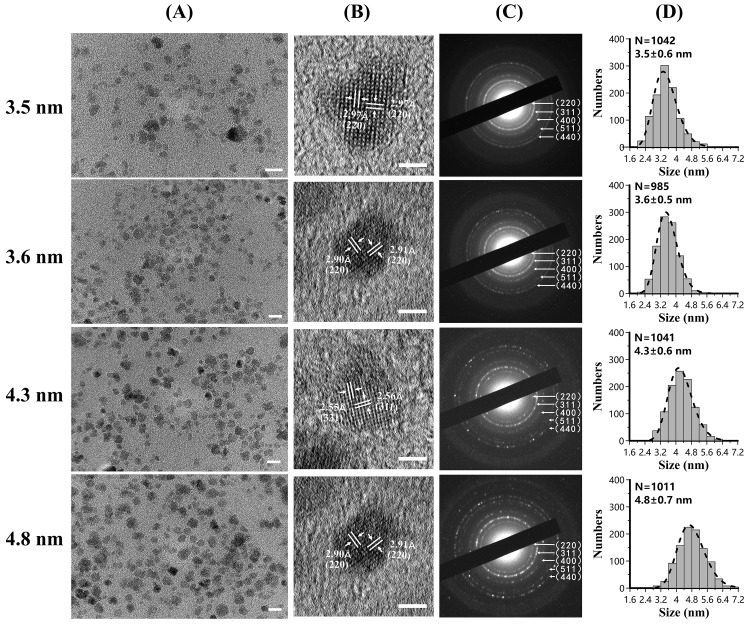
Transmission electron microscopy (TEM) characterization for synthesized MHFn nanoparticles. Column (**A**). low resolution TEM images. Scale bar: 10 nm. Column (**B**). high resolution TEM images. Scale bar: 2 nm. Column (**C**). selected area electron diffraction images. Column (**D**). size distribution. N, number of particles measured. Dotted line is the lognormal distribution fitting.

Low magnification TEM micrographs (Figure 1A) show that the MHFn nanoparticles were well dispersed. The HR-TEM micrographs (Figure 1B) show the clear lattices fringes without obvious lattice defects. The lattice fringes measured from HR-TEM micrographs matched with magnetite and/or maghemite. The diffraction rings in SAED pattern images (Figure 1C) were clear and sharp, which indicates the magnetic cores were crystalline. The mean size of MHFn cores are 3.5 ± 0.6 nm, 3.6 ± 0.5 nm, 4.3 ± 0.6 nm, 4.8 ± 0.7 nm (Figure 1D and Table 1), respectively, with a lognormal distribution (dotted lines).

### 3.2. Magnetic Properties

**Figure 2:**
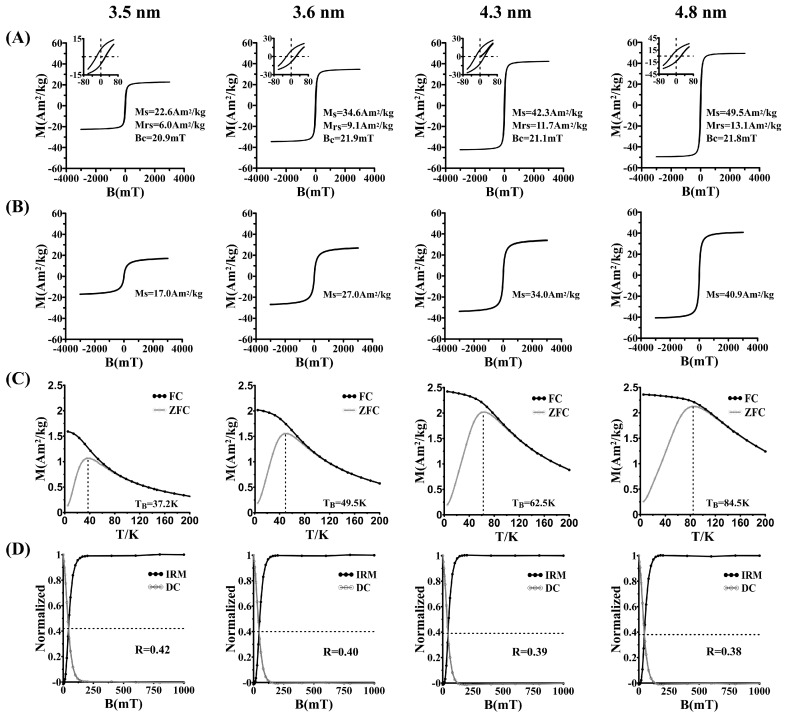
Magnetism characterization for synthesized MHFn nanoparticles. Row (**A**). hysteresis loops measured at 5 K. Subgraph is an enlargement of the field at ±80 mT. Row (**B**). hysteresis loops measured at 300 K. Row (**C**). ZFC/FC magnetization curves. Row (**D**). normalized IRM acquisition and DC demagnetization of SIRM measured at 5 K.

The saturation magnetization (Ms) measured at 5 K (Figure 2A) and 300 K (Figure 2B) and blocking temperature (T_B_) (Figure 2C) increased with increasing MHFn core size. The decrease of R-value (Figure 2D) suggests that the static magnetic interaction is slightly increased with core size. At 300 K, the magnetization hysteresis loops M (H) curves show that all MHFn nanoparticles are superparamagnetic (no measurable coercivity). The magnetic data is summarized in Table 1.

**Figure 3:**
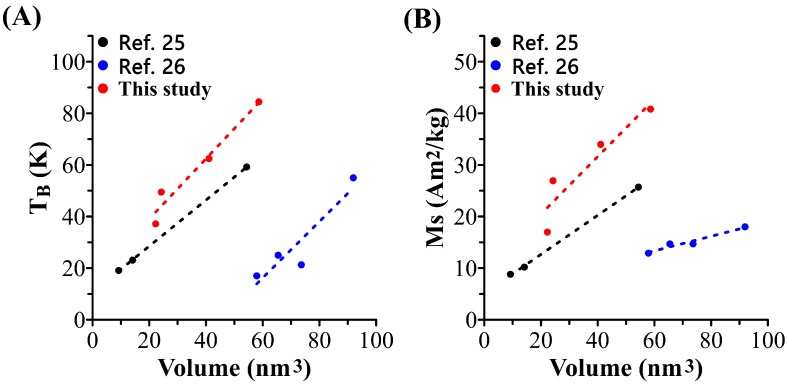
(**A**) influence of nanoparticle volumes on blocking temperature (T_B_) and (**B**) saturation magnetization (Ms) (300 K). Dotted line is the linear fitting of the data.

Interestingly, the T_B_ increases linearly with the volume of MHFn (Figure 3A) in this study and our previous study [25,26]. The slope may be used to roughly calculate the effective anisotropic energy constant K_eff_ (k_eff_V = 25k_B_T_B_) [27]. The Ms measured at 300 K likely increases linearly with the volume of measured samples (Figure 3B).

### 3.3. Dynamic Light Scattering (DLS) Measurements and Thermogravimetric (TG) Analysis

**Figure 4:**
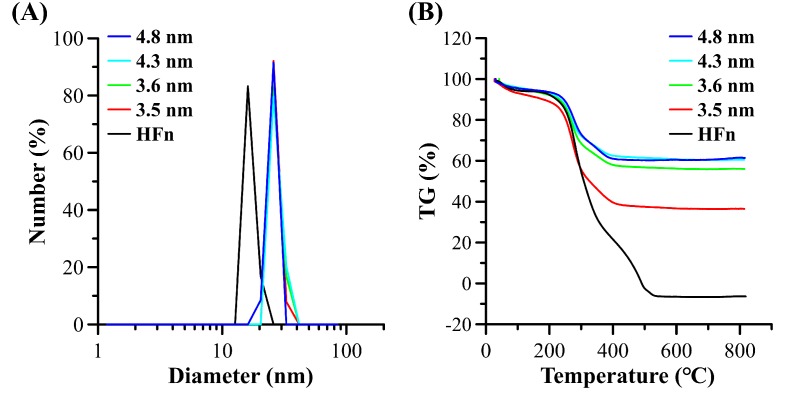
Dynamic light scattering (**A**) and Thermogravimetry (**B**) characterization of MHFn nanoparticles.

The hydrodynamic diameter of pure HFn is about 16 nm while the mineralized MHFn is about 26 nm, which is larger than the HFn (Figure 4A, Table 1). The inner mineral core may be responsible for this gap. As expected, the inner core mass proportion of particles was increased as core size increases (Figure 4B and Table 1). A clear weight loss from 200–300 °C may be related to protein shell loss.

### 3.4. Specific Loss Power and Intrinsic Loss Power

#### 3.4.1. Stability of MHFn Nanoparticles in Aqueous

**Figure 5:**
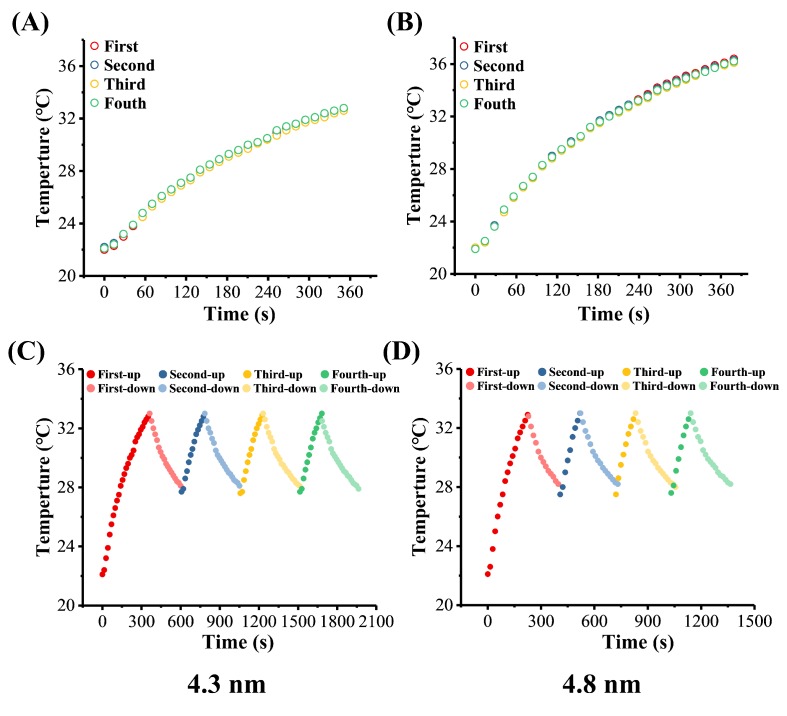
Repeatability of heating efficiency of the MHFn in aqueous under AMF treatment. (**A,B**) Single measurement of temperature rise for four times for 4.3 and 4.8 nm samples, respectively. (**C,D**) Continuous measurement of temperature rise on heating to 33 °C and cooling to 28 °C for four cycles. All the measurements were performed at c = 5 mg[Fe]/mL, magnetic field H = 19.5 kA/m and *f* = 805.5 kHz.

Samples with mean size of 4.3 nm and 4.8 nm were used for analysis. As shown in Figure 5, temperature rising curves of both 4.3 nm and 4.8 nm MHFn were all well overlapped by the single test method (Figure 5A,B), and the same periodical changes were obtained during the four cycles by continuous test method (Figure 5C,D) for both 4.3 nm and 4.8 nm MHFn. The results indicated that the MHFn are stable in aqueous medium during the AMF treatment.

#### 3.4.2. Core-Size and Fe Concentration Effects

**Figure 6:**
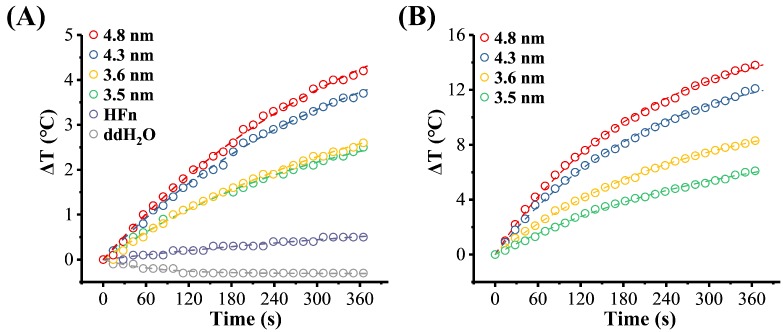
Heating efficiency of MHFn nanoparticles with different core sizes at (**A**) 1.5 mg[Fe]/mL and (**B**) 5 mg[Fe]/mL. All the measurements were performed in a magnetic field H = 19.5 kA/m and *f* = 805.5 kHz. Dotted lines are the fitting results of Box-Lucas equation.

Figure 6 shows the effects of core-size and Fe concentration on the temperature rise of MHFn samples. In this study, two Fe concentrations were used, 1.5 mg[Fe]/mL (Figure 6A) and 5 mg[Fe]/mL (Figure 6B). The SLPs fitting results have been summarized in Table 2. The temperature rise (∆T) at 6 min increases from 2.5 to 4.2 °C with core size increases at 1.5 mg[Fe]/mL (Figure 6A and Table 2), while increases from 6.1 to 13.8 °C at 5 mg[Fe]/mL (Figure 6B and Table 2).

**Figure 7:**
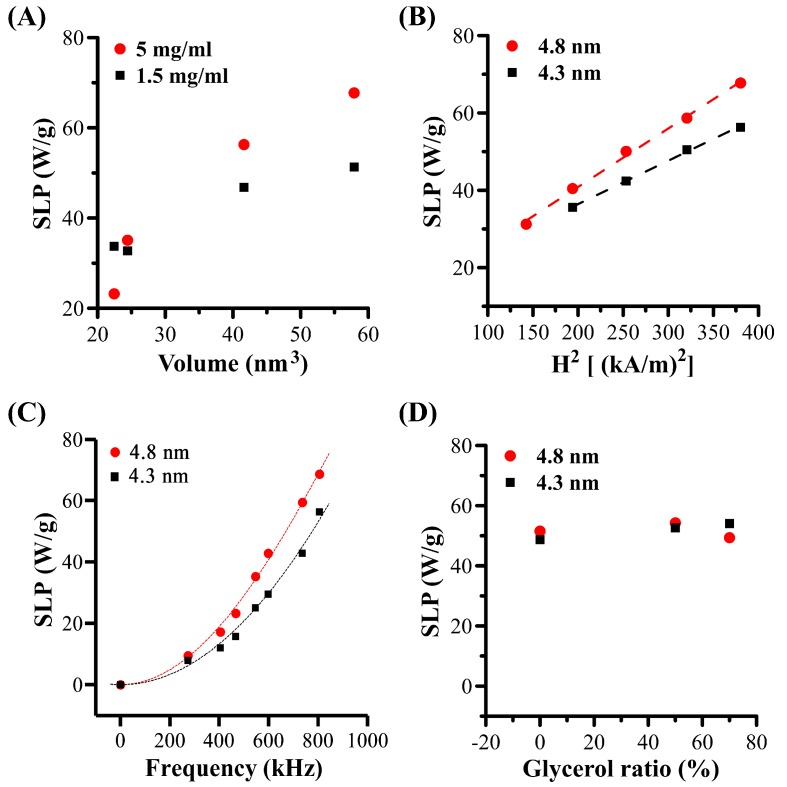
The Special Loss Power (SLP) vs. different factors. (**A**) SLP vs. MHFn particles volume. Measurements were performed at H = 19.5 kA/m and *f* = 805.5 kHz. (**B**) SLP vs. amplitude. Measurements were performed at c_(4.8 nm)_ = c_(4.3 nm)_ = 5 mg[Fe]/mL, *f* = 805.5 kHz. Dotted lines were linear fitting with SLP = a*f*H^2^ + b. (**C**) SLP vs. frequency. Measurements were performed at c_(4.8 nm)_ = c_(4.3 nm)_ = 5 mg[Fe]/mL, H = 19.5 kA/m. Lines were represent fits results of equation (1). (**D**) SLP vs. glycerol ratio. Measurements were performed at c_(4.8 nm)_ = 1.5 mg[Fe]/mL, c_(4.3 nm)_ = 2.5 mg[Fe]/mL, H = 19.5 kA/m and *f* = 805.5 kHz.

SLP also increases from 32.7 to 51.3 W/g[Fe] at 1.5 mg[Fe]/mL (Figure 7A and Table 2) and from 23.2 to 67.7 W/g[Fe] at 5 mg[Fe]/mL (Figure 7A and Table 2). 

Our results show that SLPs of MHFn samples were increased with increasing Fe concentration. However, previous works have shown that SLPs were increased [28,29] or decreased [30,31] with the increasing Fe or nanoparticle concentration. Furthermore, some works have shown that SLPs were independent of Fe or nanoparticle concentration [12,32]. These inconsistent findings may indicate a complicated mechanism of Fe concentration effects on SLP for different MNPs. Nevertheless, for most MNPs, it is believed that increasing the concentration will increase the dipolar interaction while increasing the magnetic anisotropy energy, which results in the SLP increasing gradually to a maximum and then starting to decrease [33]. Generally, as the concentration increases, SLP increases for the smaller particles and decreases for the lager particles with the increasing Fe concentration [34]. Studies on MHFn samples in this work suggest the increasing of SLP with increasing Fe concentration to some extent.

#### 3.4.3. Magnetic Field Effects and the Néel Relaxation

**Figure 8:**
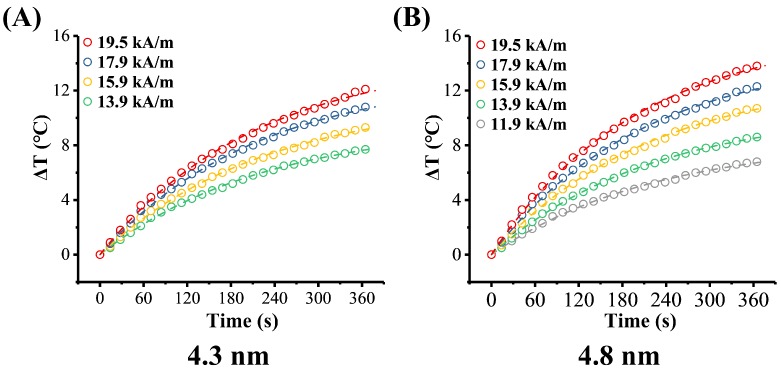
Magnetic field amplitude dependence on heating efficiency of 4.3 nm (**A**) and 4.8 nm (**B**) MHFn nanoparticle samples at 5 mg[Fe]/mL. All the measurements were performed at 805.5 kHz. Dotted lines are the fitting results of Box-Lucas equation.

Due to the better temperature rising effect of 4.3 nm and 4.8 nm sized MHFn, we mainly focused on this two samples in the following studies. The effect of the AMF parameter on temperature rise of 4.3 nm and 4.8 nm MHFn samples were carried out at 5 mg[Fe]/mL in aqueous medium. As AMF amplitude increases from 11.9 to 19.5 kA/m under a frequency of 805.5 kHz, the ∆T of 4.3 nm MHFn at 6 min increased from 7.7 to 12.0 °C (Figure 8A and Table 2), and from 6.8 to 13.8 °C for 4.8 nm (Figure 8B and Table 2), respectively. SLPs also increase from 35.6 to 56.3 W/g for 4.3 nm MHFn and from 31.2 to 67.8 W/g for 4.8 nm MHFn (Figure 7B and Table 2). Based on linear response theory (LRT), we fitted the SLP vs. amplitude data with the equation SLPs = a*f*H^2^ + b and found that the data were well characterized with a R^2^ > 0.99 (Figure 7B). Note that the slope *a* is directly the ILP value proposed by Kallumadil [24].

**Figure 9:**
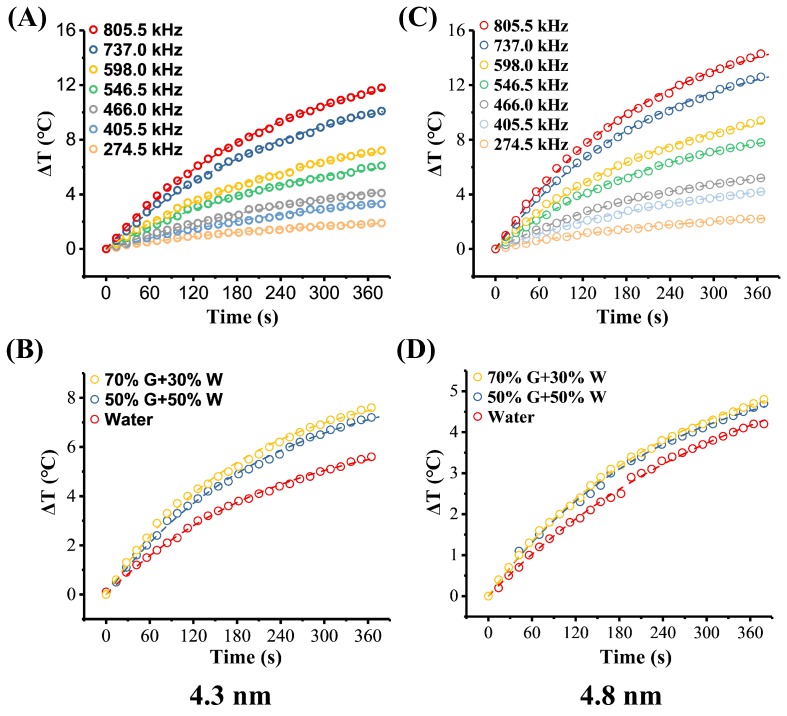
Effects of magnetic field frequency and viscosity on heating efficiency of 4.3 nm (**A**,**B**) and 4.8 nm (**C**,**D**) samples. Dependence of AMF frequency on heating efficiency of (**A**) 4.3 nm and (**C**) 4.8 nm MHFn nanoparticles with concentration of 5 mg[Fe]/mL, measured at 19.5 kA/m. Temperature rise measurement in different glycerol-water mixture of (**B**) 4.3 nm sample at 2.5 mg[Fe]/mL and (**D**) 4.8 nm sample at 1.5 mg[Fe]/mL performed at magnetic field H = 19.5 kA/m and *f* = 805.5 kHz. Dotted lines are the fitting results of Box-Lucas equation.

Figure 9A,B and Table 2 show the effect of AMF frequency on temperature rise for this two MHFn samples. The ΔT at 6 min increased about six-fold (from 1.9 °C to 11.5 for 4.3 nm MHFn and from 2.2 to 14.2 °C for 4.8 nm MHFn) when AMF frequency increases from 274.5 to 805.5 kHz under amplitude 19.5 kA/m (Figure 9A,B). It was noted that the SLP also significantly increased with increasing frequency for both 4.3 nm (from 7.9 to 56.3W/g) and 4.8 nm (from 9.5 to 68.6W/g) samples (Figure 7C and Table 2). Here, we fitted the SLPs vs. frequency curve with the formula below [12]:

(1) $$SLP\left(f\right)=A\times \frac{\tau_{fit}\times {\left(2\pi f\right)}^{2}}{1+{\left(\tau_{fit}\times 2\pi f\right)}^{2}}$$

where *A* is a parameter about the magnetic property of sample, *τ_fit_* is fitted effective relaxation time. As shown in Figure 7C, the data were well described by the equation with R^2^ > 0.99. The fitted relaxation time τ_fit_ were shown in Table 3.

The theoretical Néel and Brownian relaxation timew were calculated with the equations developed by Rosensweig [6]. Comparing the relaxation time between fitted relaxation time (τ_fit_), calculated Néel relaxation time (τ_N_), Brownian relaxation time (τ_B_) and effective relaxation time τ_eff_ (Table 3), we found both τ_eff_ and τ_fit_ is comparable to τ_N_.

Additionally, we also measured the temperature rise of MHFn samples in different glycerol ratio solutions (Figure 9B for 4.3 nm MHFn and Figure 9B for 4.8 nm MHFn) to simulate different viscosities. Although the coefficients of viscosity were increased from 1.0 to 22.5 mPa‧s (at 20 °C) [35], SLPs were basically unchanged for both 4.3 nm and 4.8 nm samples (low than 10%) (Figure 7D and Table 2), which is obviously different from previous studies where the SLPs would significantly decrease with increasing viscosity based on a Brownian mechanism [10,36]. Combined the results of theoretical calculated relaxation time and actual measurements under different viscosity conditions, we propose that heat generation mechanism for both 4.3 nm and 4.8 nm MHFn samples is dominated by Néel relaxation.

#### 3.4.4. Intrinsic Loss Power (ILP)

**Figure 10:**
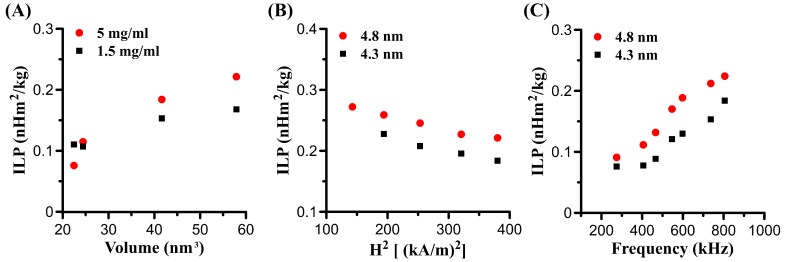
The intrinsic loss power (ILP) vs. different factors. (**A**) ILP vs. particles volume. Measurements were performed at H = 19.5 kA/m and *f* = 805.5 kHz. (**B**) ILP vs. frequency. Measurements were performed at c_(4.8 nm)_ = c_(4.3 nm)_ = 5 mg[Fe]/mL, H = 19.5 kA/m. (**C**) ILP vs. amplitude. Measurements were performed at c_(4.8 nm)_ = c_(4.3 nm)_ = 5 mg[Fe]/mL, *f* = 805.5 kHz.

Like SLP, ILP increases with size or volume and increasing Fe concentration (Figure 10A and Table 2). Figure 10C shows the effect of AMF frequency on ILP. We note that the ILP increases with increasing frequency, which indicates that SLPs are not linear with frequency for the measured samples, consistent with a previous study [37]. However, ILP may be independent of frequency for those MNPs whose SLPs are linear with frequency [38].

As we mentioned in Section 3.4.3, ILP can be obtained by fitting SLPs vs. amplitude. We found the ILP decreases with increasing amplitude for both 4.3 nm and 4.8 nm MHFn samples (Figure 10B), which is consistent with previous studies [39,40]. Nevertheless, in the formula ILP = SLP/*f*H^2^, if we consider the intercept b in SLP (SLP = a*f*H^2^ + b), the ILP could be calculated with ILP = (a*f*H^2^ + b)/*f*H^2^ = a + b/*f*H^2^. Obviously, the relationship between ILP and amplitude is closely related to the value of intercept b. If b = 0, ILP is independent of amplitude; if b > 0, ILP will decrease with increasing amplitude; oppositely, if b < 0, ILP will increase with increasing amplitude. In this work, ILP decreases with increasing amplitude, and the reason may be the positive intercept b obtained for 4.3 nm MHFn (b = 14.0) and for 4.8 nm MHFn (b = 10.6). Considering these effects on ILP, it may not advisable to calculate the ILP of the particles directly under a single AMF parameter test.

### 3.5. Physical Model

The equation of motion for spherical MHFn particles in a viscous medium is given by:

(2) $$I\dot{\omega }=f_{r}\omega +\mu_{0}\mu \times H+\xi_{B}$$

where *I* is the moment of inertia, ω is the angular velocity vector, *f_r_* is the rotational viscous friction coefficient, μ is the magnetic moment, H is the applied field, and ξ_B_ is the random perturbation torque associated with Brownian motion. The maximum magnitude of the magnetic torque relative to the viscous torque can be evaluated assuming $f_{r}=8\pi \eta a_{h}^{3}$ for a sphere with hydrodynamic radius ah in a medium with dynamic viscosity *η*, and $\mu =4\pi \mu_{s}a_{m}^{3}/3$ for the magnetic moment of the MHFn’s magnetic core with spontaneous magnetization *μ_s_* and radius *a_m_*. Equating ω=2πf with the frequency of the applied field, the ratio between magnetic and viscous torques becomes:

(3) $$\frac{\xi_{m}}{\xi_{h}}\leq \frac{\mu_{0}\mu_{s}H}{12\pi \eta f}\times {\left(\frac{a_{m}}{a_{h}}\right)}^{3}$$

Near-unit values are obtained with maghemite-like (i.e., *μ*_s_ = 380 kA/m), uncoated (i.e., *a_m_ = a_h_*) particles in water (*η* = 0.001 Pa⋅s) at the lowest frequency (274.5 kHz) used in these experiments. However, the much smaller magnetic core in MHFn ensures that the magnetic torque is at least two orders of magnitude smaller than the viscous drag, meaning that the magnetic moment is affected only by Néel’s relaxation in the applied field, as for with static magnetic measurements of Figure 2 for mechanically blocked particles.

Heat generation is thus related to the energy loss ∆*U* of the hysteresis loop *M(H)* of mechanically blocked particles by:

(4) $$P=f\Delta U=-\mu_{0}\oint M\left(H\right)dH$$

where the integral represents the area enclosed by the loop. It is therefore possible to compare Equation (4) with the generated heat power if room-temperature hysteresis loops measured at the same frequency and same maximum field as the heat-generating field were available. Because the hysteresis opening is proportional to the amount of blocked particles, one can use Néel’s magnetic relaxation theory to find measurement time *τ* and temperature *T* combinations that yield the same fraction of blocked particles and thus the same hysteresis opening. These combinations obey the simple relation:

(5) $$\frac{T_{2}}{T_{1}}=\frac{\ln\left(\tau_{1}/\tau_{0}\right)}{\ln\left(\tau_{2}/\tau_{0}\right)}$$

with *τ*_0_ ≈ 0.1 ns being the atomic reorganization time. Using *T*_1_ ≈ 300 K and $\tau_{1}={\left(2\pi f\right)}^{-1}$ for the room-temperature heating experiments, one gets the temperature:

(6) $$T_{2}=-T_{1}\frac{\ln\left(\tau_{0}f\right)}{\ln\left(\tau_{2}/\tau_{0}\right)}$$

of equivalent static hysteresis measurements such as those of Figure 2, where *τ*_2_ is the total time needed for the measurements. For instance, *f* = 805.5 kHz and *τ*_2_ = 6 h (the typical measurement time required by a MPMS SQUID magnetometer) yields *T*_2_ = 85 K. A hysteresis measurement performed at this temperature using the same maximum field *H*_0_ of the heating experiments can be used to estimate the hysteresis loss power with Equation (4), after correcting for the temperature dependence of *Ms*, that is:

(7) $$p\left(T_{1},H_{0}\right)=-\mu_{0}f\frac{M_{S}\left(T_{1}\right)}{M_{S}\left(T_{2}\right)}\oint M\left(H,T_{2},H_{0}\right)dH$$

This model will likely explain the dependence of SLP on $H_{0}^{2}$ (the opening of Rayleigh loops is proportional to $H_{0}^{2}$), as well as the quadratic dependence of SLP on *f*, since the loop opening depends on the fraction of blocked particles, which increases at larger frequencies.

## 4. Conclusions

Based on this study, the following conclusions can be drawn:

(1) The heating efficiency SLP of MHFn nanoparticles increased with the core-size, Fe concentration, AMF frequency, and amplitude.
(2) The fitted relaxation time τ_fit_ and calculated effective relaxation time τ_eff_ were closer to calculated the Néel relaxation time τ_N_, and the SLP of 4.3 nm and 4.8 nm MHFn basically unchanged in different viscosity glycerol-water mixtures. This indicates that the AMF heating generation mechanism of MHFn nanoparticles is dominated by Néel relaxation.
(3) The calculated ILP of MHFn nanoparticles under different AMF frequency and amplitude conditions varied.
(4) The max temperature rise on 6 min measured is 14.2 °C for 4.8 nm MHFn at 5 mg[Fe]/mL under 805 kHz and 19.5 kA/m. This suggests that MHFn nanoparticles may be applied to medical treatments in future for tumor magnetic hyperthermia, heat-triggered drug release with consideration of good biocompatibility and active targeting to various tumors.

## Figures and Tables

**Table 1:** Core size, magnetic properties of the synthesized MHFn nanoparticles.

Sample	Core Size (nm)	D_h_(PDI) ^a^ (nm)	T_B_ (K)	R value	5 K-Hysterisis		300 K-Hysterisis	TG (%)
					Ms (Am^2^/kg)	Bc (mT)	Ms (Am^2^/kg)	
A	3.5 ± 0.6	16.8(0.10)	37.2	0.42	22.6	20.9	17.0	36.5
B	3.6 ± 0.5	26.5(0.07)	49.5	0.40	34.6	21.9	27.0	56.1
C	4.3 ± 0.6	27.1(0.10)	62.5	0.39	42.3	21.1	34.0	60.6
D	4.8 ± 0.7	25.4(0.06)	84.5	0.38	49.5	21.8	40.9	61.5

^a^ D_h_: hydrodynamic diameter, PDI: polydispersity index.

**Table 2:** Summary of temperature rise, SLP and ILP under different Fe concentration, medium and AMF parameter measurement condition.

Core Size (nm)	Fe Concentration (mg[Fe]/mL)	Solution	Frequency (kHz)	Amplitude (kA/m)	ΔT at 6 mins (°C)	SLP (W/g[Fe])	ILP (nHm^2^/kg)
4.8	1.5	Water	805.5	19.5	4.2	51.3	0.17
4.3	1.5	Water	805.5	19.5	3.7	46.8	0.15
3.6	1.5	Water	805.5	19.5	2.6	32.7	0.11
3.5	1.5	Water	805.5	19.5	2.5	33.7	0.11
4.8	5	Water	805.5	19.5	13.8	67.7	0.22
4.3	5	Water	805.5	19.5	12.0	56.3	0.18
3.6	5	Water	805.5	19.5	8.2	35.1	0.11
3.5	5	Water	805.5	19.5	6.1	23.2	0.08
4.3	5	Water	805.5	13.9	7.7	35.6	0.23
4.3	5	Water	805.5	15.9	9.2	42.4	0.21
4.3	5	Water	805.5	17.9	10.7	50.5	0.20
4.3	5	Water	805.5	19.5	12.0	56.3	0.18
4.8	5	Water	805.5	11.9	6.8	31.2	0.27
4.8	5	Water	805.5	13.9	8.5	40.4	0.26
4.8	5	Water	805.5	15.9	10.7	50.1	0.25
4.8	5	Water	805.5	17.9	12.3	58.7	0.23
4.8	5	Water	805.5	19.5	13.8	67.8	0.22
4.3	5	Water	274.5	19.5	1.9	7.9	0.08
4.3	5	Water	405.5	19.5	3.3	12.0	0.08
4.3	5	Water	466.0	19.5	4.0	15.7	0.09
4.3	5	Water	546.5	19.5	6.0	25.1	0.12
4.3	5	Water	598.0	19.5	7.0	29.5	0.13
4.3	5	Water	737.0	19.5	9.9	42.9	0.15
4.3	5	Water	805.5	19.5	11.5	56.3	0.18
4.8	5	Water	274.5	19.5	2.2	9.5	0.09
4.8	5	Water	405.5	19.5	4.2	17.2	0.11
4.8	5	Water	466.0	19.5	5.2	23.3	0.13
4.8	5	Water	546.5	19.5	7.8	35.7	0.17
4.8	5	Water	598.0	19.5	9.3	42.9	0.19
4.8	5	Water	737.0	19.5	12.6	59.4	0.21
4.8	5	Water	805.5	19.5	14.2	68.6	0.22
4.3	2.5	**100% water** + 0 glycerol	805.5	19.5	5.5	48.7	0.16
4.3	2.5	**50% water** + 50% glycerol	805.5	19.5	7.2	52.6	0.17
4.3	2.5	**30% water** + 70% glycerol	805.5	19.5	7.6	54.1	0.18
4.8	1.5	**100% water** + 0 glycerol	805.5	19.5	4.2	51.5	0.17
4.8	1.5	**50% water** + 50% glycerol	805.5	19.5	4.6	54.3	0.18
4.8	1.5	**30% water** + 70% glycerol	805.5	19.5	4.7	49.3	0.16

**Table 3:** Calculated relaxation time.

Sample	τ_B_ (s)	τ_N_ (s)	τ_eff-cal_	τ_fit_
4.3 nm	6.5 × 10^−6^	7.3 × 10^−8^	7.2 × 10^−8^	1.1 × 10^−8^
4.8 nm	6.5 × 10^−6^	4.0 × 10^−7^	3.8 × 10^−7^	7.5 × 10^−8^
